# Generating Bessel beams with broad depth-of-field by using phase-only acoustic holograms

**DOI:** 10.1038/s41598-019-56369-z

**Published:** 2019-12-27

**Authors:** Sergio Jiménez-Gambín, Noé Jiménez, José M. Benlloch, Francisco Camarena

**Affiliations:** Instituto de Instrumentación para Imagen Molecular, Consejo Superior de Investigaciones Científicas, Universitat Politècnica de València, Camino de Vera S/N, 446022 València, Spain

**Keywords:** Acoustics, Metamaterials

## Abstract

We report zero-th and high-order acoustic Bessel beams with broad depth-of-field generated using acoustic holograms. While the transverse field distribution of Bessel beams generated using traditional passive methods is correctly described by a Bessel function, these methods present a common drawback: the axial distribution of the field is not constant, as required for ideal Bessel beams. In this work, we experimentally, numerically and theoretically report acoustic truncated Bessel beams of flat-intensity along their axis in the ultrasound regime using phase-only holograms. In particular, the beams present a uniform field distribution showing an elongated focal length of about 40 wavelengths, while the transverse width of the beam remains smaller than 0.7 wavelengths. The proposed acoustic holograms were compared with 3D-printed fraxicons, a blazed version of axicons. The performance of both phase-only holograms and fraxicons is studied and we found that both lenses produce Bessel beams in a wide range of frequencies. In addition, high-order Bessel beam were generated. We report first order Bessel beams that show a clear phase dislocation along their axis and a vortex with single topological charge. The proposed method may have potential applications in ultrasonic imaging, biomedical ultrasound and particle manipulation applications using passive lenses.

## Introduction

Bessel functions are exact and invariant solutions of the Helmholtz equation^1,2^, i.e., an ideal Bessel beam do not experience diffraction. These beams present remarkable properties as self-healing, beam-width close to the diffraction limit and excellent depth-of-field. In addition, high-order Bessel beams present phase dislocations, i.e., they conform vortex beams and transport orbital angular momentum. First proposed by Durnin in 1987^1^, Bessel beams have been broadly studied in both, optics^1–6^ and acoustics^7–9^. In the particular case of acoustics, they have found practical applications in ultrasound imaging systems^10–14^. Their long depth-of-field and narrow beam-width allows an accurate scanning of the transmitted beam, while their self-healing properties grant remarkable robustness to tissue scattering and its diffraction-free properties offer an almost constant imaging resolution with depth. Moreover, in recent years vortex beams and, in particular, Bessel beams, have attracted attention due to their special properties for particle trapping, manipulation or rotation applications^15–25^ or acoustic radiation force applications in fluids^26^. Vortex beams have also been proposed for robust acoustic communications^27,28^.

**Figure 1:**
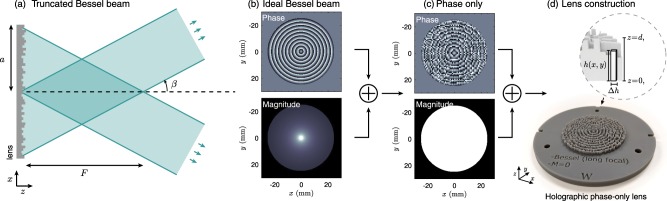
(**a**) Scheme of a truncated Bessel beam for a monochromatic wave. Lens generation process: (**b**) Phase and magnitude of an ideal acoustical axicon, (**c**) resulting phase and magnitude after processing, (**d**) manufactured holographic phase-only lens and its geometrical definition.

An ideal Bessel beam is generated by a converging conical wavefront of infinite extent. When it converges to the axis of symmetry and interferes with itself, the tilted conical wavefront generates the characteristic Bessel beam pattern. Thus, Bessel beams in the far-field are characterized by a single-ring pattern arising from their narrow angular spectrum. However, to generate ideal Bessel beams an infinite amount of energy is needed. In practice, truncated Bessel beams are generated by using finite-aperture sources or lenses, as shown in Fig. 1(a).

In acoustics, there exist many methods to generate zero-th and high-order Bessel beams. While, as mentioned, they cannot be ideally generated in practice, several methods have been proposed to generate truncated Bessel beams. One simple strategy is to use a circular slit: at the axis, a Bessel beam pattern is generated, in analogy with optics^2^. However, the small aperture strongly limits the transmitted energy. Bessel beams were efficiently generated using arrays of annular active piezoelectric sources^10–12,22,29–32^. In this cases, each of the annular active elements is set according to a Bessel function in both, phase and amplitude. By realising the similarity between the Bessel function and the radial modes of piezoelectric sources this process can be mimicked using simple discs, though the radiation efficiency is low^33^.

Beyond active arrays of transducers, Bessel beams can also be obtained by coupling a passive lens to a single active source. One example are acoustical axicons^34,35^, in analogy with optical ones^36,37^. Acoustic axicons are conical-shaped lenses: when waves are refracted along their tilted surface a conical wavefront is generated. A variation of axicons are fraxicon lenses^38^. Fraxicons, in analogy to Fresnel lenses, are stepped phase plates able to produce a similar conical wavefront due to the refraction along its sawtooth-shaped profile. In this way, the thickness of the lens can be strongly reduced^39^.

Instead of using refraction processes, Bessel beams can be obtained by using the diffraction of waves on axisymmetric gratings^40^. These lenses produce Bessel beams in a broad range of frequencies with frequency-dependent properties. Other approaches include the combination of a focused source with a layered structure^41^. The main drawback of these approaches is that the resulting beams could present aberrations due to multiple reflections inside the bulk of the lenses. Finally, metasurfaces have been proposed for generation of zero-th order Bessel beams using the reflection of sound waves in air^42^, but their performance is usually limited to a very narrow frequency band as they are based in local resonances.

Acoustic beams can carry phase dislocations producing acoustic vortices^43^. In these conditions the beam carries pseudo-angular momentum^18^, as occurs in the case of high-order Bessel beams. High-order Bessel beams can be analogously generated using Archimedes’ spiral gratings where the order of the Bessel beam, i.e., the topological charge of the beam, is proportional to the number of arms of the spiral^44–48^. Equivalently, active elements with spiral geometries can also generate high-order Bessel beams^49,50^. The phase rotation needed to generate high-order Bessel beams can be generated using phase plates^51,52^. Developable helicoidal/conical air-coupled active surfaces with phase dislocations have been also proposed using curved piezoceramics^53^ or flexible ferroelectrets^54^. Finally, recently metasurfaces have been proposed to engineer the phase to generate acoustic vortices^55–58^.

While the field distribution in the transverse direction of the beam produced by these methods is accurately described by a Bessel function, all *passive* methods present a common drawback: the axial distribution of the field is not constant as required by the Bessel beam solution initially proposed by Durnin. In this way, the field-of-view is limited and these beams present a focal spot. In particular, using the cited passive methods to generate the beam, its intensity grows roughly linear with space. This is a geometric consequence of the axisymmetric converging wavefront: to maintain constant the field along the axis the energy contained in all annular regions of the circular aperture should be constant.

In this work, we present a simple method to generate zero-th and high-order Bessel beams of flat-intensity along their axis using phase-only acoustic holograms. These kind of lenses have recently been proposed to generate arbitrary acoustic fields and, simultaneously, to correct the strong aberrations during the propagation of transcranial ultrasound both with phase-and-amplitude encoding^59^ or only with phase encoding^60,61^. Other applications of holographic phase-only lenses include particle manipulation applications^62^, multi-frequency focal generation^63^ or photoacoustic generation of complex holographic fields^64^. Magnitude-and-phase lenses based on metamaterials have been proposed to control sound waves in air^65^. However, their applicability in the field of biomedical ultrasound is still very limited mainly due to the deep-subwavelength geometrical features of the resulting structures. In addition, most of the designs for metamaterials in air, where the solid phase can be considered perfectly rigid in most cases, are not directly applicable in biomedical ultrasound because it must be included the coupling between the solid metamaterial structure and either water or water-like tissues. Acoustic holograms present a robust and simple approach for these applications. Recently, phase and amplitude holograms were developed by using two phase holograms to produce in-plane acoustic images^66^. However, in practice stationary waves between the two holograms can degrade the image. In this work, we encode both phase and magnitude information into a single phase-only holographic lens. Using this approach, we experimentally, numerically and theoretically report the generation of zero-th and high-order Bessel beams with elongated field-of-view (of about 40 wavelengths) in the ultrasound regime.

## Flat-Intensity Bessel-Beams Design

We consider a truncated Bessel beam where the converging conical wavefront is tilted an angle *β* with respect to the normal. If the beam is generated using a source of aperture 2*a*, the non-diffracting beam extends, in an ideal situation, from *z* = 0 to $z=F=(a/2)\tan \,\beta$, as shown in Fig. 1(a). To generate a particular complex wavefront at the source plane (*z* = *d*), as the one shown in Fig. 1(c), we employ a holographic phase-plate that can be manufactured using 3D printing techniques, as shown in Fig. 1(d). These phase plates provide a robust way to engineer the phase-and-amplitude distribution along the surface of the plane^59,62^.

The lens surface was divided in squared pixels of different height, *h*(*x*,*y*), and uniform width, Δ*h*, as shown in Fig. 1(d). We assume each elastic column to vibrate longitudinally as a Fabry-Pérot resonator. For each column, the field at the holographic plane located at ${{\bf{x}}}_{0}=(x,y,d)$ is given by the complex transmission coefficient^67^:

1 $$T({{\bf{x}}}_{0})=\frac{2Z{{\rm{e}}}^{-i{k}_{0}[d-h({{\bf{x}}}_{0})]}}{2Z\,\cos [{k}_{L}h({{\bf{x}}}_{0})]+i({Z}^{2}+1)\sin \,[{k}_{L}h({{\bf{x}}}_{0})]},$$

where *d* is the distance from the bottom of the lens (*z* = 0) to the holographic surface, the normalized impedance is given by $Z={Z}_{L}/{Z}_{0}$, and ${Z}_{0}={\rho }_{0}{c}_{0}$ is the impedance of water and ${Z}_{L}={\rho }_{L}{c}_{L}$, ${k}_{L}=\omega /{c}_{L}$, ${\rho }_{L}$ and *c*_*L*_, are the impedance, wavenumber, density and sound speed of the lens material. To invert Eq. (1), we used numerical interpolation. In this way, by tuning the height of each Fabry-Pérot resonator, the phase at the output of each pixel can be tailored to that of a target holographic surface.

The transmission coefficient is close to one if low relative impedance materials are used to build the phase plates, as it is the case of photosensitive polymers used for stereolithographic 3D printing. In this case, to tailor the complex holographic field (with a particular phase and amplitude as shown in Fig. 1(b)) to an equivalent phase-only field (as shown in Fig. 1(c)), we make use of a direct conversion method^68^. The basis of this direct method is the sequential scanning of the pixels to modify the complex transmission coefficient. Extended details can be found in the Methods section.

## Results

**Figure 2:**
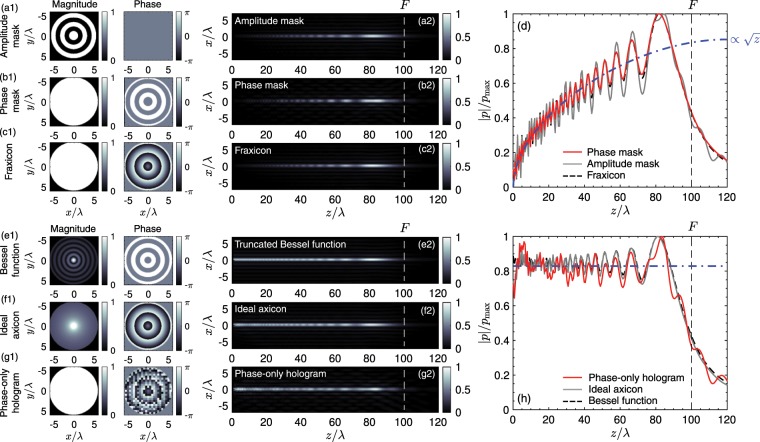
Different methods to generate a Bessel beam, calculated theoretically. (a1) Magnitude and phase distribution at the source of an axisymmetric diffraction grating (amplitude mask type). (a2) Resulting axial pressure field distribution (normalized). (b1) Magnitude and phase distribution at the source of an axisymmetric diffraction grating (phase mask type) and (b2) corresponding axial pressure field distribution. (c1) Magnitude and phase distribution at the source of an acoustical fraxicon (a steeped axicon) and (c2) corresponding axial pressure field distribution. (d) Axial pressure for the non-flat axial intensity Bessel beams. (e1) Magnitude and phase distribution at the source of an exact truncated Bessel function. (e2) Resulting axial pressure field distribution (normalized). (f1) Magnitude and phase distribution at the source of an ideal axicon (f2) corresponding axial pressure field distribution. (g1) Magnitude and phase distribution at the source of a phase-only acoustical hologram lens encoded with the ideal axicon and (g2) its corresponding axial pressure field distribution. (h) Axial pressure for the flat-axial-intensity Bessel beams.

The initial conditions corresponding to common approaches to produce zero-th order Bessel beams are shown in Fig. 2(a1–c1). First, we show the Bessel beam produced by a binary-amplitude grating^40^, which is equivalent to a Soret-type Zone Plate lens^69^. This lens is composed of alternating axisymmetric opaque and transparent zones separated a distance *b*, as shown in Fig. 2(a1). Due to the continuity of the wave-vector at the interface, the propagating wavefront presents a phase distribution of

2 $${p}_{0}(r)=\exp (i{k}_{r}r),$$

where ${k}_{r}=\,\sin (\beta ){k}_{0}$ is the transverse wave-vector, fixed by the lens, ${k}_{0}=\omega /{c}_{0}$ the wavenumber and $\beta ={\sin }^{-1}(2\pi /{k}_{0}b)$ is the angle of tilted wavefront of the Bessel beam. While a Bessel beam is generated^40^ its axial field distribution is not constant, as shown in Fig. 2(a2). As the total surface of each annular area varies with the radial coordinate, the intensity radiated by each annulus depends linearly with the distance to the centre. Therefore, the intensity pattern grows linearly with distance (and proportional with the square root of the axial distance, see Fig. 2(d)). An analogous situation occurs when the lens is an alternating phase-inversion axisymmetric grating, as shown in Fig. 2(b1). This can be obtained by using a phase plate where the thicknesses vary between 2 values, $L=[(2m-1)\lambda /2,m\lambda ]$ with $m\in {\mathbb{N}}$. In this case the resulting beam intensity also grows linearly with the axial distance as shown Fig. 2(b2). Equivalently for axisymmetric gratings, acoustical axicons (cone-shaped lenses which have been proposed for optics and acoustics) are also capable of producing a converging conical wavefront due to refraction on its tilted surface. A variation of conical axicon lenses are fraxicon lenses. Fraxicons, in analogy to Fresnel lenses, present a wrapped profile able to produce a similar tilted wavefront by the refraction along its inclined surface. The phase profile of a fraxicon varies linearly with the radial coordinated as ${p}_{0}(r)=\exp (i{k}_{r}r)$, as shown in Fig. 2(c1). However, the exterior concentric annular areas radiate more energy as compared with the interior ones as they present more surface, as occurs with the axisymmetric gratings In this way, the corresponding axial field distribution is not constant either, as shown in Fig. 2(d).

In contrast, the field at the source plane of an ideal (truncated) Bessel beam given by

3 $${p}_{0}(r)={J}_{n}({k}_{r}r),$$

where *J*_*n*_ is the *n*-th order Bessel beam, is shown in Fig. 2(e1). The resulting field distribution is shown in Fig. 2(e2,h), showing an almost flat profile. Here, the beam extends from $z=0$ to $z=F$, a total distance of 100 *λ*. The existing ripples are caused by the limited aperture of the source. Note that the source pattern presents both, magnitude and phase variations, which are of the order of the wavelength. It is worth noting here that to our knowledge, the source condition corresponding to Eq. (3) has only been reproduced using active methods using a finite set of concentric rings.

In order to reduce the complexity of the Bessel pattern one can design an equivalent field distribution using an ideal axicon, i.e., imposing a compensation for the intensity as a function of the radial coordinate. By simple geometrical considerations, this results in a source field of

4 $${p}_{0}(r)=\frac{1}{\sqrt{{\rm{\max }}(r,{r}_{0})}}\exp (i{k}_{r}r),$$

where *r*_0_ is set to a finite value to avoid the singularity, e.g., the pixel size, as shown in Fig. 2(f1). This field distribution also produces a Bessel beam of constant amplitude analogously to that of the ideal-truncated Bessel function, as demonstrated in Fig. 2(f2,h). Note the ripples match those of the ideal-truncated Bessel function. However, the source field for the ideal axicon presents a reduced spatial complexity as compared with the ideal Bessel function. This makes it more suitable to apply a direct method to obtain an equivalent phase-only lens. Once applying the direct method^68^, the resulting phase-only hologram is shown in Fig. 2(g1), using pixel size of $\lambda /8$. The field generated by this lens is shown in Fig. 2(g2,h). We can observe that the field distribution is flat and the characteristic ripples of the Bessel beam obtained by using the hologram agree with those of the finite-aperture Bessel beam.

### Performance of holographic bessel beams

**Figure 3:**
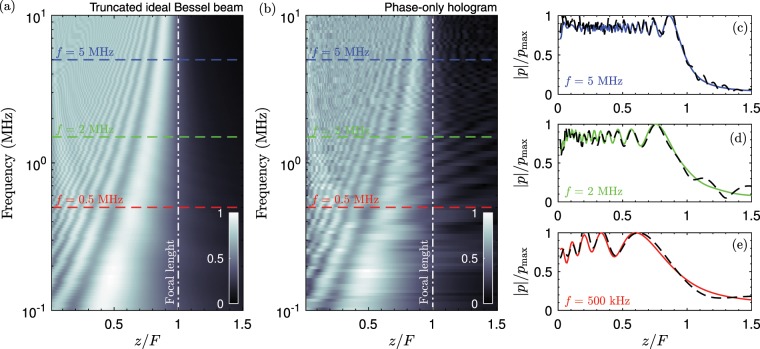
Axial field distribution as a function of the design frequency for (**a**) a truncated-ideal Bessel beam and (**b**) a phase-only hologram calculated using the Rayleigh-Sommerfeld integral. (**c**) Axial field distribution for a Bessel beam of *f* = 5 MHz (continuous-blue) and the corresponding field produced by the phase-only hologram (dashed). (**d**) Axial field distribution for a Bessel beam of *f* = 2 MHz (continuous-green) and the corresponding field produced by the phase-only hologram (dashed). (**e**) Axial field distribution for a Bessel beam of *f* = 500 kHz (continuous-red) and the corresponding field produced by the phase-only hologram (dashed).

Diffraction effects of any beam are stronger for low frequencies, e.g., when the ratio $a/\lambda$ is small. Thus, the capability of any finite-aperture Bessel beam to concentrate the acoustic energy over the axis will be also limited by the natural diffraction of the beam. We have designed 100 holograms for frequencies ranging from 100 kHz to 10 MHz in order to study the effect of the frequency in their focusing performance. The aperture of the source and the focal length of the lens were maintained constant (*a* =  25 mm and *F* = 50 mm). Figures 3(a,b) show the axial field distribution for the ideal truncated Bessel beam and the corresponding Bessel beam generated by the phase-only hologram. First, we can observe that both calculations agree, showing that the proposed method can be used in a broad range of frequencies. In particular, Figs. 3(c,d,e) show the axial distribution for 0.5 MHz, 2 MHz and 5 MHz. These frequencies correspond to $a/\lambda \approx (8,33,83)$, respectively. We can observe that for higher frequencies the spatial distribution shows a flatter profile and the energy corresponding to both, ideal and holographic Bessel beams is evenly distributed along the axis. For lower frequencies, e.g., at $f=0.5$ MHz, both beams suffer from stronger diffraction effects and, therefore, the energy of the beam only extends to a fraction of the focal length (up to $z\approx 0.8F$ in this particular case). In addition, at lower frequencies the amplitude of the ripples is increased as compared with the ones corresponding to higher frequencies. Note that this behaviour is not a limitation of the method using holograms, it is caused by the natural diffraction of the wavefront and it will be observed in any truncated Bessel beam propagating in homogeneous and linear media.

### Broadband behaviour of fraxicons and phase-only holograms

In the previous section we have shown how fraxicons and holograms perform at the design frequency. In this section, we design a fraxicon and a hologram for a particular frequency and then evaluate their performance for other excitation frequencies. We might notice that the field distributions at the source for both fraxicon and ideal Bessel beams present spatial modulations in the radial direction characterized by the distance $b=2\pi /{k}_{r}$, with the transverse wavenumber fixed by ${k}_{r}={k}_{0}\,\sin (\beta )$ (see e.g. the concentric rings separated a distance *b* that appears at the source distributions shown in Fig. 2). When a wave impinges these lenses its phase and amplitude is modified according to the local thickness of the lens. For frequencies much lower than the design frequency (*λ* > *b*), a small phase change is produced because the maximum height of the fraxicon lens is much lower than the wavelength. Therefore, the field is transmitted and the field of a piston-like source is retrieved. However, for frequencies lower than the design frequency but higher than $\lambda \le b$, waves are diffracted due to the spatial modulation of the lens, even when the transmitted phase modulation is small. In this situation, fraxicons and holograms act in a similar manner than axisymmetric diffraction gratings^40^.

Due to the conservation of the transverse wavenumber at the boundary, waves are diffracted at angles given by ${\beta }_{n}(\omega )={\sin }^{-1}(n\lambda /b)$ producing a tilted conical wavefront for each diffraction order *n*. In consequence, the extent of the Bessel beam changes with the excitation frequency. By simple trigonometric relations we can obtain the focal length for each diffraction order as

5 $${F}_{n}(\lambda )=\frac{ab}{n\lambda }\sqrt{1-{(\frac{n\lambda }{b})}^{2}}.$$

Note that for *λ >*  *b* the focal distance becomes imaginary for all diffraction orders. This implies a lower cut-off frequency for the fraxicons and phase-only holograms, *f*_low_, which is fixed by the lens design as

6 $${f}_{{\rm{l}}{\rm{o}}{\rm{w}}}=\frac{{c}_{0}}{b}={f}_{0}\,\sin (\beta )={f}_{0}\frac{a}{\sqrt{{a}^{2}+{F}^{2}}}.$$

Below this frequency phase-only holograms and fraxicons whose maximum height corresponds to *λ* cannot produce any Bessel beam.

**Figure 4:**
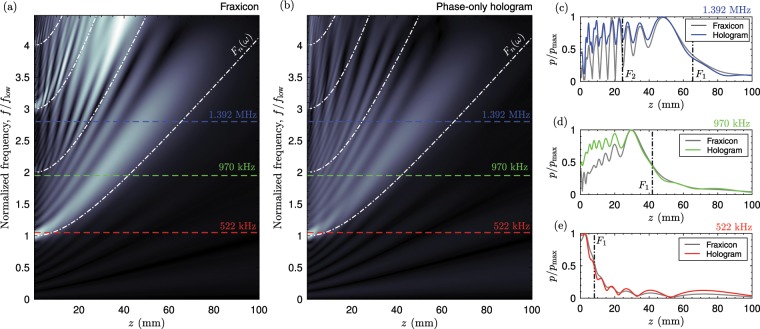
Axial field distribution for a zero-th order Bessel beam as a function of the excitation frequency of (**a**) a fraxicon and (**b**) phase-only hologram of *F* = 50 mm, *a* = 25 mm and for a design frequency of *f*_0_ =  1.112 MHz. The focus of the *n*-th diffraction order, *F*_*n*_, is shown in dotted-dashed white lines. (**c**–**e**) Axial field distributions at 1.392 MHz, 970 MHz and 522 kHz, respectively. The focus of the *n*-th diffraction order, *F*_*n*_, is shown in dotted-dashed black lines.

Figure 4 shows the axial field distribution of a zero-th order Bessel beam as a function of the frequency for a fraxicon (Fig. 4(a)) and phase-only hologram (Fig. 4(b)) with the design parameters *F* = 50 mm, *a* = 25 mm and *f*_0_ = 1.112 MHz. This results in a ring distance of *b* = 3 mm, with imposes the lower cut-off frequency of *f*_low_ = 497.3 MHz. The focus of the *n*-th diffraction order, *F*_*n*_, is shown in dotted-dashed white lines. We can see that the focal length is extended from *z* = 0 to $z={F}_{n}$, with *n* = 1. Some energy, is also transmitted for frequencies below $\lambda  > b$, corresponding to the zero-th diffraction order. For frequencies around $\lambda \approx b$ the focal length of the first diffraction order is close to the source (e.g., *F*_1_ = 8 mm at 522 kHz). Therefore, the field is strongly focused at this distance, as shown in Fig. 4(e). As the phase-only hologram maintain a similar spatial modulation with characteristic rings separated a distance *b*, a similar focusing pattern is observed, as shown in Fig. 4(b,e). For higher frequencies, e.g., at 970 kHz, the focus is extended to *F*_1_ = 41.9 mm. Accordingly, the field distributes up to this distance. On the one hand, the axial field distribution of the fraxicon is distributed with a square root dependence of the distance (its intensity is linear with the distance). On the other hand, the hologram produces a uniform field distribution, as shown in Fig. 4(d). Finally, for higher frequencies high-order diffraction order appear, each one presenting a different focal length given by Eq. (5). Each diffraction order produces a new Bessel beam that is overlaps with the preceding one. An example is given in Fig. 4(c) for *f* = 1.392 MHz. The field produced by the first diffraction order extends up to *F*_1_ = 65.4 mm, while the focus of the second diffraction order appears at *F*_2_ = 24.5 mm. The coherent sum of the two fields produce an interference pattern with strong axial variations between $z=0$ and $z={F}_{2}$. Overall, the phase-only hologram produces a flatter beam because the spatial modulations corresponding to rings separated a distance *b* are smoothed by the error-diffusion algorithm.

### Pixel quantization

In standard stereolithographic 3D printing techniques, the lenses are manufactured using layers of finite thickness. In addition, in simulations the pixels are represented with a discrete set of heights. In particular, in the simulations included in this paper the pixels are discretized in 221 *μ*m steps (the grid resolution), and for the experiments in 100 *μ*m steps (the printer resolution). The corresponding phase distributions of the fraxicons and holograms are therefore quantized in discrete steps.

**Figure 5:**
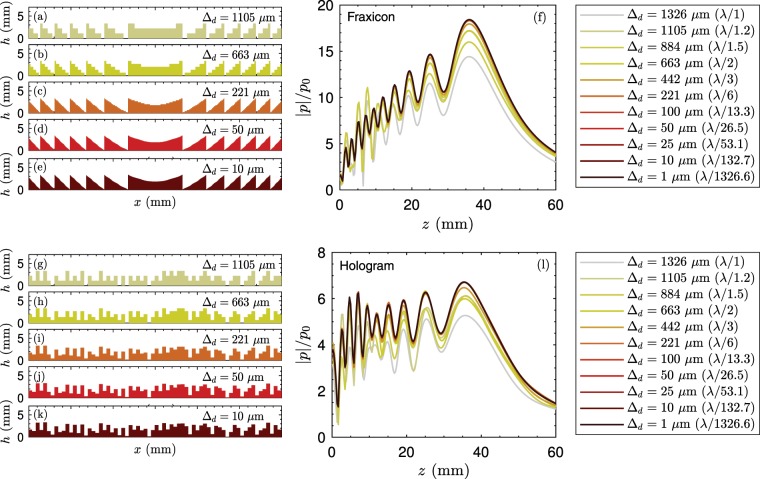
(**a**–**e**) Transversal cut of the fraxicons using different discretization heights. (**f**) Corresponding theoretical field for the fraxicons using different discretization heights. (**g**–**k**) Transversal cut of the phase-only holograms using different discretization heights. (**l**) Corresponding theoretical field for the phase-only holograms using different discretization heights.

To test the impact of the height quantization we have designed a set of fraxicons and holograms using different quantization steps (Δ_*d*_), ranging from Δ_*d*_ = 1 *μ*m to Δ_*d*_ = 1.3 mm, as shown in Fig. 5. The phase is calculated using Eq. (1) and the acoustic field was obtained by using the Rayleigh-Sommerfeld equation (see Methods section). Several fraxicons were designed using different quantization levels, as shown in Fig. 5(a–e). The axial field for each fraxicon is shown in Fig. 5(f). First, the shape of the pressure distribution for all the fraxicons roughly agree the one corresponding to the lower quantitation level. Even for the cases when only 6 values are available for the pixel height (Δ_*d*_ ≈ 663 *μ*m), the steeped lenses produce similar fields. Note the case of using only two values for the height corresponds to an axisymmetric phase grating, which can also produce a Bessel beam. One remarkable difference is that when using coarser quantization levels, the amplitude of the field is reduced. This is caused because energy is diffracted into higher diffraction orders, causing secondary focal spots near the source. The quantization level used in the simulations (Δ_*d*_ = 221 *μ*m) produces a field in excellent agreement with the one corresponding to Δ_*d*_ = 1 *μ*m, showing that the accuracy of the simulated (and manufactured) lenses is enough to produce Bessel beams with flat pressure distributions. The same study was extended to phase-only holograms using different quantization levels, as shown in Fig. 5(g–k). The corresponding axial field for each phase-only hologram is shown in Fig. 5(l). We observe that phase-only holograms using low quantization levels, e.g. Δ_*d*_ < 100 *μ*m, are able to produce fields which are in close agreement with the one corresponding to Δ_*d*_ = 1 *μ*m. Moreover, quantization levels of only ${\Delta }_{d}\approx \lambda /3$ are enough to produce fields in agreement with the truncated-Bessel beam distributions. The main difference is that, as observed previously in fraxicons, at lower quantization levels energy can be diffracted into higher diffraction orders and, therefore, the gain of the lens $(|{p}_{max}|/{p}_{0})$ is reduced.

Finally, it is worth to mention here that the gain of fraxicon lenses is higher (about 2.74 times) than the gain of phase-only lenses. The reduction of the gain is caused because holograms produced by the error-diffusion algorithm mimic the phase-and-magnitude distribution of ideal Bessel beams, which presents lower amplitude at the external areas of the lens (see Fig. 2(g1).

### Experimental validation

**Figure 6:**
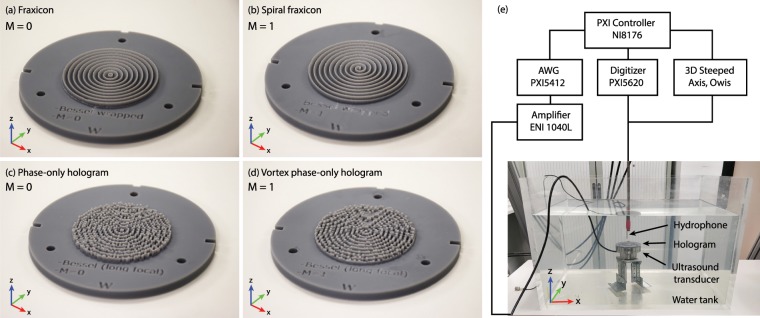
Manufactured lenses by 3D printing. (**a**) Fraxicon for a zero-th order Bessel beam (*M* = 0), (**b**) Spiral fraxicon for a first order Bessel beam (*M* = 1), (**c**-**d**) Phase-only holographic lenses for flat-intensity Bessel beams corresponding to *M* = 0 and *M* = 1 (vortex). (**e**) Diagram of the experimental set-up and photograph of one experiment in water tank.

We performed an experimental test in the ultrasonic regime using a phase-only hologram of aperture 2*a* = 50 mm and a depth-of-field *F* = 50 mm (≈40*λ*) using a source of 1.11 MHz in water. In addition, a full-wave simulation was performed using a time-domain pseudo-spectral method with *k*-space correction. A fraxicon also was manufactured for comparison. Both zero-th and first order Bessel beams were considered. The 3D-printed lenses are shown in Fig. 6(a–d) and the experimental setup is shown Fig. 6(e). Details about the measurement procedures and simulation methods can be found in the Methods section.

#### Zero-th order Bessel beams

**Figure 7:**
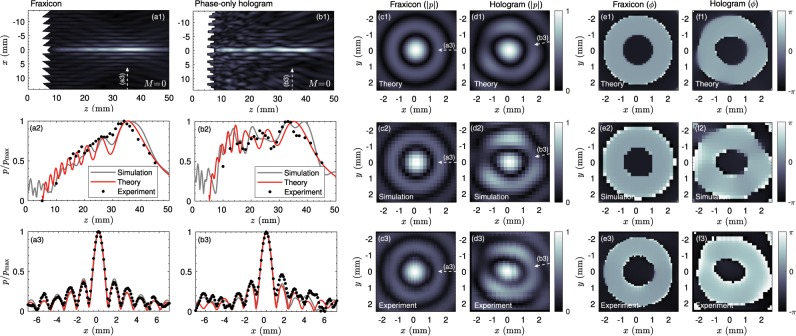
Results for the zero-th order Bessel beams. Field distribution in the (a1) sagittal plane, (a2) axial direction and (a3) transverse direction (at *z* = 35 mm) for the fraxicon lens. Field distribution in the (b1) sagittal plane, (b2) axial direction and (b3) transverse direction (at *z* = 35 mm) for the phase-only hologram. (c1–c3) Field magnitude (|*p*|) in the transverse direction (at *z* = 35 mm) for the fraxicon obtained using theory (c1), simulations (c2) and experiments (c3) for the fraxicon. (d1–d3) Corresponding field in the transverse direction for the phase-only hologram. Phase of the field (*ϕ*) in the transversal direction for (e1–e3) the fraxicon and (f1–f3) phase-only hologram.

The simulated field distribution of the fraxicon is shown in Fig. 7(a1), showing a non-flat field distribution as discussed theoretically earlier. The simulated and experimental axial field distribution, shown in Fig. 7(a2), agree with those obtained by theory. While the transversal field distribution measured at *z* = 35 mm for the fraxicon matches that of a truncated Bessel beam (see Fig. 7(a3)), the axial field distribution grows proportionally to the square root of the axial distance.

The corresponding simulated field of the phase-only hologram is shown in Fig. 7(b1). We can observe that the field distribution is remarkably uniform as compared with the fraxicon. The axial field distribution is shown in Fig. 7(b2) where, again, simulated and experimental data are in reasonable agreement with theory. Some discrepancies are observed due to the finite size of the pixel used, which is restricted by the 3D-printing technology (the lateral resolution of the printer is 50 *μ*m).

#### High-order bessel beams (vortex beams)

Beyond zero-th order, Bessel beams of higher order containing phase dislocations can also be generated by the proposed technique. We manufactured a phase-only hologram for a first-order Bessel beam using a pressure distribution of

7 $${p}_{0}(r,\theta )=\frac{1}{\sqrt{{\rm{\max }}(r,{r}_{0})}}\exp (i{k}_{r}r)\exp (iM\theta ),$$

where the topological charge of a first-order Bessel beam corresponds to *M* = 1 and *θ* is the polar angle. A spiral fraxicon, corresponding to a blazed-vortex axicon, was also manufactured for comparison using a source profile of

8 $${p}_{0}(r,\theta )=\exp (i{k}_{r}r)\exp (iM\theta ).$$

**Figure 8:**
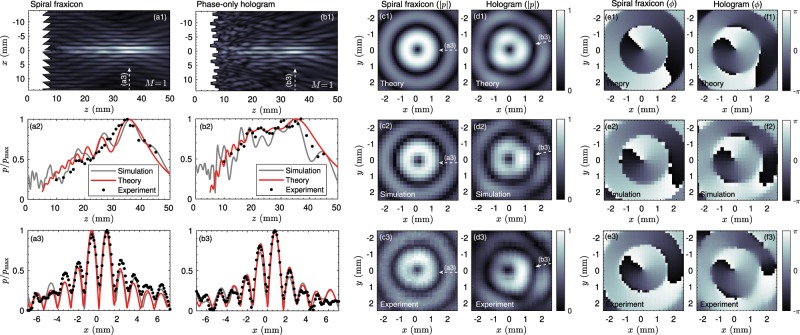
Results for the first order Bessel beams, i.e., vortex beams. Field distribution in the (a1) sagittal plane, (a2) axial direction and (a3) transverse direction (at *z* = 35 mm) for the spiral fraxicon lens. Field distribution in the (b1) sagittal plane, (b2) axial direction and (b3) transverse direction (at *z* = 35 mm) for the phase-only vortex hologram. (c1–c3) Field magnitude (|*p*|) in the transverse direction (at *z* = 35 mm) obtained using theory (c1), simulations (c2) and experiments (c3) for the spiral fraxicon. (d1–d3) Corresponding field in the transverse direction for the phase-only hologram. Phase of the field (f) in the transversal direction for (e1–e3) the spiral fraxicon and for (f1–f3) the phase-only hologram.

Figure 8(a1) shows the simulated field for the spiral fraxicon while Fig. 8(b1) shows the corresponding one for the phase-only hologram. As occurs with the zero-th order Bessel beam, our approach results in a flatter beam profile. The corresponding axial and lateral cross-sections are given in Fig. 8(a2,b2,a3,b3), respectively. The measured and simulated pressure distributions agree with the theoretical ones. The hollow beam is reproduced by all methods with the characteristic null at the centre. This null arises because of the phase dislocation of the vortex. The magnitude and phase transversal field distributions are shown in Fig. 8(a4,a5,b4,b5), respectively. We can see that the magnitude of the field shows a good symmetry in both cases, while a phase proportional to $\exp (iM\theta )$ is reproduced. In all cases, the topological charge of the vortex is the unity (*M* = 1) while at *r* = 0 a phase dislocation is observed.

## Conclusions

We have presented a simple method to generate Bessel beams of flat intensity by using acoustic holograms. The magnitude and phase distribution of an ideal axicon was transformed into an equivalent phase-only hologram applying a direct method, and used to manufacture an ultrasonic lens by using 3D printing. We demonstrated theoretically the approach, and validated it by using full-wave simulations and experiments. In particular, a zero-th order Bessel beam was first produced in the ultrasonic regime. The beam evenly distributes the acoustic energy along a flat line along an axial distance of 50 mm (≈40λ) while the width of the beam remained constant at about 1 mm $(\approx \,0.7\lambda )$. Using the proposed approach, the generated beam pattern match those of an ideal (truncated) Bessel beam. Note that, in contrast, traditional methods using passive ultrasonic devices produce non-uniform beam patterns.

Beyond zero-th order beams, we have demonstrated that higher-order Bessel beams can also be generated. A first order Bessel beam was obtained, where the characteristic vortex containing a phase dislocation was observed experimentally in excellent agreement with theory and full-wave simulations. It is worth noting here that, in contrast with zero-th order Bessel beams, higher-order Bessel beams cannot be generated by using radially symmetric active systems: active transducers with chiral symmetry are required making this approach a robust solution to generate flat-intensity beams with broad depth-of-field. We remark that in contrast to Bessel beams produced by computer-generated holograms in optics^70,71^, the axial intensity of the beam using the present approach is flat.

The proposed method opens the pathway to future investigations of the singular properties of Bessel beams including their nonlinear propagation features^18,72,73^, angular momentum transfer^18^ and particle manipulation capabilities^15,19,74–78^. These beams may find potential uses in particle manipulation and acoustic radiation force techniques, ultrasound imaging or therapeutic ultrasound applications.

## Methods

### Analytical field calculation: Rayleigh-sommerfeld diffraction integral

For theoretical calculations we use a semi-analytical method using Rayleigh-Sommerfeld diffraction integral. The acoustic pressure field given by *p*(**r**) at point **r**, generated by a moving surface *S* of arbitrary shape located at coordinates **r**_0_ and vibrating with a complex particle velocity ${v}_{0}({{\bf{r}}}_{0})$ normal to the surface, is given by the Rayleigh-Sommerfeld diffraction integral as^79^:

9 $$p({\bf{r}},\omega )=\frac{i\omega {\rho }_{0}}{2\pi }{\int }_{S}\frac{{v}_{0}({{\bf{r}}}_{0})\exp (-i{k}_{0}|{\bf{r}}-{{\bf{r}}}_{0}|)}{|{\bf{r}}-{{\bf{r}}}_{0}|}dS,$$

where $\omega =2\pi f$; ${k}_{0}=\omega /{c}_{0}$, ${c}_{0}$ and ${\rho }_{0}$ are, respectively, the wavenumber, sound speed and density of water. The hologram is included in the analytical calculations by setting a source field distribution as ${v}_{0}({{\bf{r}}}_{0},\omega )=T({{\bf{r}}}_{0},\omega ){p}_{0}/{\rho }_{0}{c}_{0}$, where $T({{\bf{r}}}_{0},\omega )$ is the transmission coefficient for each hologram pixel given by Eq. (1), and ${p}_{0}$ is the pressure amplitude exerted by the piezoelectric source, that was set to 1 Pa to match simulations.

### Numerical field calculation: Pseudo-spectral time-domain simulations

For numerical calculations we use a pseudo-spectral simulation method with *k*-space dispersion correction to numerically integrate the linearised constitutive relations of acoustics^80,81^. In an inhomogeneous and absorbing media, the governing equations, i.e., the continuity equation, the momentum conservation equation and the pressure-density relation, can be written as three-coupled first-order partial differential equations as:

10 $$\frac{\partial \rho ^{\prime} }{\partial t}=-\,\rho \nabla \cdot {\bf{u}}-{\bf{u}}\cdot \nabla \rho ,$$

11 $$\frac{\partial {\bf{u}}}{\partial t}=-\,\frac{1}{\rho }\nabla p,$$

12 $$p={c}^{2}(\rho ^{\prime} +{\bf{d}}\cdot \nabla \rho -{\rm{L}}\rho ^{\prime} ),$$

where ${\bf{u}}={\bf{u}}({\bf{r}},t)$ is the acoustic particle velocity, ${\bf{d}}={\bf{d}}({\bf{r}},t)$ is the acoustic particle displacement, $p=p({\bf{r}},t)$ is the acoustic pressure, $\rho ^{\prime} =\rho ^{\prime} ({\bf{r}},t)$ is the acoustic density, $\rho =\rho ({\bf{r}})$ is the ambient (or equilibrium) density, $c=c({\bf{r}})$ is the sound speed, and $L=L({\bf{r}},t)$ is a linear operator introducing the frequency-dependent absorption and dispersion^80^. Absorption following a power-law on frequency given by $\alpha ({\bf{r}},\omega )={\alpha }_{0}({\bf{r}}){\omega }^{\gamma }$, where ${\alpha }_{0}({\bf{r}})$ is the absorption coefficient and *γ* is the exponent of the frequency power law, together with its corresponding physical dispersion are included by the integro-differential operator as:

13 $${\rm{L}}=\tau \frac{{\rm{\partial }}}{{\rm{\partial }}t}{(-{{\rm{\nabla }}}^{2})}^{\frac{\gamma }{2}-1}+\eta {(-{{\rm{\nabla }}}^{2})}^{\frac{\gamma +1}{2}-1},$$

where $\tau =-\,2{\alpha }_{0}{c}^{\gamma -1}$ and $\eta =2{\alpha }_{0}{c}^{\gamma }\,\tan (\pi \gamma /2)$ are the absorption and dispersion proportionality coefficients. This operator is solved efficiently using the fractional Laplacian in the *k*-space. With this method we can approximate numerically the wave propagation through inhomogeneous and lossy media, allowing the full-wave simulation of the phase plate. In particular, the hologram is represented implicitly by a heterogeneous material following the shape of the lens, i.e., $\rho ({\bf{r}})={\rho }_{L}$ and $c({\bf{r}})={c}_{L}$ for all the points **r** that belong to interior of the lens, and $\rho ({\bf{r}})={\rho }_{0}$ and $c({\bf{r}})={c}_{0}$ for all the points **r** corresponding to water. It is worth noting here that this simulation method is selected because it provides low numerical dispersion as compared with finite-differences methods^82^. We use a numerical grid with a spatial step of $\Delta x=\Delta y=\Delta z=221\,\mu {\rm{m}}$ and a numerical temporal step of $\Delta t=18.1\,{\rm{ns}}$, leading to a Courant-Friedrichs-Lewy number^80^ of 0.13 in water and a spatial sampling of 6 grid points per wavelength in water for a frequency of 1.112 MHz. These parameters are fixed in all simulations in this paper.

### Experimental field measurements

The experiments were conducted inside a 1 × 0.75 × 0.5 m^3^ water tank filled with degassed and distilled water at 22°. The ultrasonic transducer was composed by a single element circular piezoceramic crystal (PZT26, Ferroperm Piezoceramics, Denmark) mounted in a custom designed stainless-steel housing with aperture 2*a* = 50 mm. The transducer was driven with a 100 cycles sinusoidal pulse burst at a frequency of *f* = 1.112 MHz by a signal generator (PXI5412, National Instruments, USA) and amplified by a linear RF amplifier (ENI 2100, ENI, Rochester, NY). The pressure field was measured by a calibrated needle hydrophone with a 500 *μ*m active diameter (HNR-500, Onda). The hydrophone signals were acquired by a digitizer (PXI5620, National Instruments, USA) and averaged 100 times to increase the signal to noise ratio. A 3D micro-positioning system (OWIS GmbH) was used to move the hydrophone in three orthogonal directions with an accuracy of 10 *μ*m. All the signal generation and acquisition processes were based on a NI8176 National Instruments PXI-Technology controller, which also controlled the micro-positioning system. Temperature measurements were performed throughout the whole process to ensure no temperature changes of ±0.5 °C. The transverse maps were acquired in the range (*x*,*y*) = ± 3 m using a step of 0.15 mm, the transverse cross-section lines were acquired in the range *x* = ± 7 m using a step of 0.1 mm, and the axial measurements were acquired from 10 to 45 mm using a step of 1 mm.

### Phase-only encoding

We use a direct method to estimate an equivalent holographic lens of uniform field magnitude^68^. The basis of this direct method is the sequential scanning of the pixels to modify the complex transmission coefficient. The method work as follows: First, the odd and even rows are scanned from opposite directions, and a bidirectional error of the diffusion process is calculated. The magnitude of each visited pixel is forced to be a constant value while the exact phase value is preserved. The resulting error is diffused to the neighbouring pixels. Finally, the result gives a surface with a modified phase depending on the bidirectional error diffusion process^68^. The main limitation of this method is that if the pixel width is small, areas with isolated long pixels, i.e., columns, that can experience bending modes can appear. Note this does not imply that a lens cannot be designed, but the theory presented here only applies to longitudinal modes on each pixel. The width of the pixels used in this work, 2/3 times the wavelength, is thick enough to ensure that the resonance frequency of the first bending mode is far away from the first longitudinal Fabry-Pérot resonance frequency.

### Lens manufacturing

Holographic lenses and fraxicons were 3D printed using stereo lithography (SLA) techniques with a Form 2 printer (Formlabs, USA), with a resolution of 50 *μ*m and 100 *μ*m in lateral and axial directions, respectively, and using a photosensitive resin (Standard Grey, Formlabs, USA). The acoustical properties of the material were obtained experimentally using a pulse-echo technique in a test cylinder, resulting in a measured sound speed of ${c}_{L}=2440.7\,{\rm{m}}/{\rm{s}}$ and a density of ${\rho }_{L}=1162\,{\rm{kg}}/{{\rm{m}}}^{3}$, and the absorption was set to *α* = 3.06 dB/cm at 1.112 MHz, matching the reported values of similar polymers^61,62^.

## Supplementary information

Supplementary information 
