# Supplementary material for: Generating Bessel beams with broad depth-of-field by using phase-only acoustic holograms
Source: Sci Rep. 2019 Dec 27;9:20104. doi: 10.1038/s41598-019-56369-z (PMC6934506; doi:10.1038/s41598-019-56369-z)
Supplement: Supplementary file 1 — Supplementary information [file 41598_2019_56369_MOESM1_ESM.pdf]

Supplementary material for “Generating Bessel beams with broad depth-of-field by using phase-only acoustic holograms”

Sergio Jiménez-Gambín[1], Noé Jiménez[1,*], José M. Benlloch[1], and Francisco Camarena[1]

[1]Instituto de Instrumentación para Imagen Molecular, Consejo Superior de Investigaciones Científicas, Universitat Politècnica de València, Camino de Vera S/N, 446022, València, Spain.
[*]Corresponding author: nojigon@upv.es

ABSTRACT

In this document we present the supplementary material of the paper entitled “Generating Bessel beams with broad depth-of-field by using phase-only acoustic holograms”. We show the measurement of the symmetry of the beam for Fraxicon (or flat axicon) and phase-only holograms obtained theoretically, by simulation, and experimentally measured for both, $M = 0$ and $M = 1$.

Symmetry of the beam

In this section we present the supplementary material of the paper entitled “Generating Bessel beams with broad depth-of-field by using phase-only acoustic holograms”. In particular, we show the beam profiles as a function of the polar angle in order to quantify the variations of the amplitude of the side lobes. Figure 1 show the radial variations of a Fraxicon (or flat axicon) and the phase-only hologram obtained theoretically, by simulation, and experimentally measured for both, $M = 0$ and $M = 1$.

For the zero-th order ($M = 0$) Bessel beam (Figs.1(a1-a4)), the Fraxicon produces a very symmetric field, note the lenses also present radial symmetry. When the field is measured at $z = 35$ mm, the experiments maintain such a symmetry indicating the good alignment of the measurement system with respect with the lens and a symmetric vibration pattern produced by the piezoelectric source.

When the zero-th order ($M = 0$) Bessel beam of wide depth-of-field is generated using the hologram (Figs.1(b1-b4)), the side-lobes present strong variations as a function of the polar angle. Since holograms are not symmetrical, neither is the field. However, one can notice that the main lobe maintain the symmetry as it can be observed in Fig.1(b4): the elongated focal spot is circular.

The first-order Bessel beam ($M = 1$) generated using the spiral Fraxicon also presents good cylindrical symmetry. Only weak variations of the beam profile were measured between different angles. Note this apply to the absolute value of the field as the complex field of the vortex beam presents chiral symmetry.

Finally, when the first order Bessel beam is generated using the hologram, the field presents more radial variations. In this case, the variations of the peak pressure amplitude of the side lobes was $0.06 \times p_{\mathrm{max}}$ for the theory, $0.07 \times p_{\mathrm{max}}$ for the simulations and $0.16 \times p_{\mathrm{max}}$ for the experiments. Note in this case since holograms are not symmetrical, neither is the field. However, note that the circular shape of the vortex is maintained, as the distance between peaks remains constant.

References

1. Durnin, J. Exact solutions for nondiffracting beams. i. the scalar theory. *J. Opt. Soc. Am. A* **4**, 651 (1987).
2. Durnin, J., Miceli Jr, J. & Eberly, J. Diffraction-free beams. *Physical review letters* **58**, 1499 (1987).
3. Chu, X. Analytical study on the self-healing property of Bessel beam. *Eur. Phys. J. D* **66**, 259 (2012).
4. McLeod, E., Hopkins, A. B. & Arnold, C. B. Multiscale Bessel beams generated by a tunable acoustic gradient index of refraction lens. *Opt. Lett.* **31**, 3155 (2006).
5. Li, Z., Alici, K. B., Caglayan, H. & Ozbay, E. Generation of an axially asymmetric Bessel-like beam from a metallic subwavelength aperture. *Phys. Rev. Lett.* **102**, 143901 (2009).
6. Fahrbach, F. & Rohrbach, A. Propagation stability of self-reconstructing Bessel beams enables contrast-enhanced imaging in thick media. *Nat. Commun.* **3**, 632 (2011).

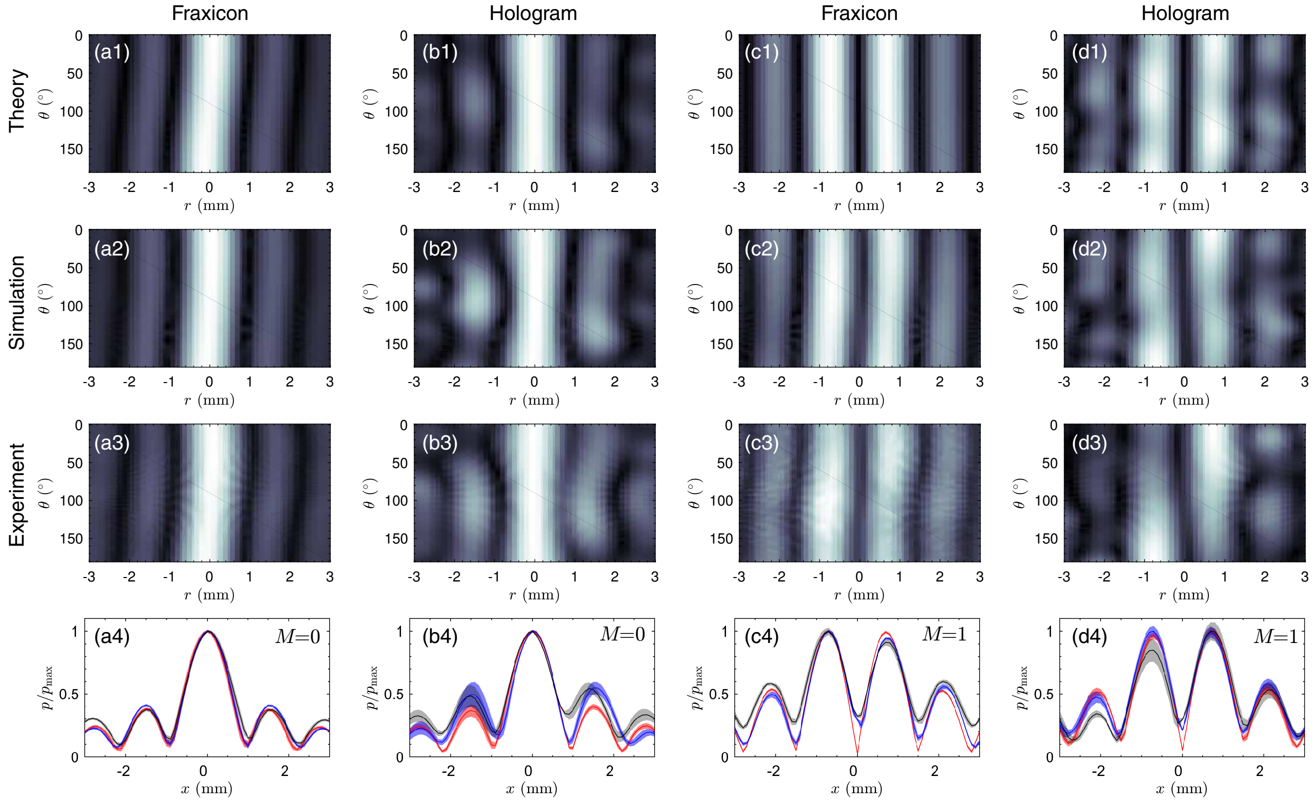

Figure 1. (a-d) Beam profiles as a function of the polar angle measured at $z = 35$ mm. (a1-a3) Theoretical, simulated and experimental beam profile for the fraxicon ($M = 0$). (a4) Radial beam profile calculated as the mean over the azimuthal angle for (red) theory, (blue) simulation and (black) experiment for the fraxicon ($M = 0$). Filled areas show the corresponding standard deviation. (b1-b3) Theoretical, simulated and experimental beam profile for the phase only hologram ($M = 0$). (b4) Radial beam profile calculated as the mean over the azimuthal angle for (red) theory, (blue) simulation and (black) experiment for the phase only hologram ($M = 0$). (c1-c3) Theoretical, simulated and experimental beam profile for the spiral fraxicon ($M = 1$). (c4) Radial beam profile calculated as the mean over the azimuthal angle for (red) theory, (blue) simulation and (black) experiment for the phase only hologram ($M = 1$). (d1-d3) Theoretical, simulated and experimental beam profile for the phase only hologram (vortex, $M = 1$). (d4) Radial beam profile calculated as the mean over the azimuthal angle for (red) theory, (blue) simulation and (black) experiment for the phase-only hologram (vortex, $M = 1$).

7. Lu, J.-y., Zou, H. & Greenleaf, J. F. Biomedical ultrasound beam forming. *Ultrasound in medicine & biology* **20**, 403–428 (1994).

8. Marston, P. L. Scattering of a Bessel beam by a sphere. *J. Acous. Soc. Am.* **121**, 753 (2007).

9. Marston, P. L. Scattering of a Bessel beam by a sphere: Ii. helicoidal case and spherical shell example. *The Journal of the Acoustical Society of America* **124**, 2905–2910 (2008).

10. Lu, J. & Greenleaf, F. Ultrasonic nondiffracting transducer for medical imaging. *IEEE Trans. Ultrason. Ferroelec. Freq. Contr.* **37**, 438 (1990).

11. Lu, J.-Y. & Greenleaf, J. F. Pulse-echo imaging using a nondiffracting beam transducer. *Ultrasound in medicine & biology* **17**, 265–281 (1991).

12. Lu, J.-y., Song, T.-K., Kinnick, R. R. & Greenleaf, J. F. In vitro and in vivo real-time imaging with ultrasonic limited diffraction beams. *IEEE transactions on medical imaging* **12**, 819–829 (1993).

13. Lu, J.-y., Xu, X.-L., Zou, H. & Greenleaf, J. F. Application of Bessel beam for doppler velocity estimation. *IEEE transactions on ultrasonics, ferroelectrics, and frequency control* **42**, 649–662 (1995).

14. Nabavizadeh, A., Greenleaf, J. F., Fatemi, M. & Urban, M. W. Optimized shear wave generation using hybrid beamforming methods. *Ultrasound in medicine & biology* **40**, 188–199 (2014).

15. Marston, P. L. Axial radiation force of a Bessel beam on a sphere and direction reversal of the force. *The Journal of the Acoustical Society of America* **120**, 3518–3524 (2006).

16. Marston, P. L. Negative axial radiation forces on solid spheres and shells in a Bessel beam. *The Journal of the Acoustical Society of America* **122**, 3162–3165 (2007).

17. Marston, P. L. Radiation force of a helicoidal Bessel beam on a sphere. *The Journal of the Acoustical Society of America* **125**, 3539–3547 (2009).

18. Thomas, J.-L. & Marchiano, R. Pseudo angular momentum and topological charge conservation for nonlinear acoustical vortices. *Physical review letters* **91**, 244302 (2003).

19. Volke-Sepúlveda, K., Santillán, A. O. & Boullosa, R. R. Transfer of angular momentum to matter from acoustical vortices in free space. *Phys. Rev. Lett.* **100**, 024302 (2008).

20. Zhang, L. & Marston, P. L. Geometrical interpretation of negative radiation forces of acoustical Bessel beams on spheres. *Physical Review E* **84**, 035601 (2011).

21. Courtney, C. R. *et al.* Dexterous manipulation of microparticles using Bessel-function acoustic pressure fields. *Applied Physics Letters* **102**, 123508 (2013).

22. Hong, Z., Zhang, J. & Drinkwater, B. W. Observation of orbital angular momentum transfer from Bessel-shaped acoustic vortices to diphasic liquid-microparticle mixtures. *Phys. Rev. Lett.* **114**, 214301 (2015).

23. Baresch, D., Thomas, J.-L. & Marchiano, R. Observation of a single-beam gradient force acoustical trap for elastic particles: Acoustical tweezers. *Phys. Rev. Lett.* **116** (2016).

24. Marzo, A., Caleap, M. & Drinkwater, B. W. Acoustic virtual vortices with tunable orbital angular momentum for trapping of mie particles. *Phys. Rev. Lett.* **120**, 044301 (2018).

25. Li, Y. *et al.* Acoustic radiation torque of an acoustic-vortex spanner exerted on axisymmetric objects. *Applied Physics Letters* **112**, 254101 (2018).

26. Riaud, A., Baudoin, M., Thomas, J.-L. & Matar, O. B. Cyclones and attractive streaming generated by acoustical vortices. *Physical Review E* **90**, 013008 (2014).

27. Shi, C., Dubois, M., Wang, Y. & Zhang, X. High-speed acoustic communication by multiplexing orbital angular momentum. *Proceedings of the National Academy of Sciences* **114**, 7250–7253 (2017).

28. Jiang, X., Liang, B., Cheng, J.-C. & Qiu, C.-W. Twisted acoustics: metasurface-enabled multiplexing and demultiplexing. *Advanced Materials* **30**, 1800257 (2018).

29. Hsu, D., Margetan, F. & Thompson, D. O. Bessel beam ultrasonic transducer: fabrication method and experimental results. *Appl. Phys. Lett.* **55**, 2066 (1989).

30. Campbell, J. A. & Soloway, S. Generation of a nondiffracting beam with frequency-independent beamwidth. *The Journal of the Acoustical Society of America* **88**, 2467–2477 (1990).

31. Masuyama, H., Yokoyama, T., Nagai, K. & Mizutani, K. Generation of Bessel beam from equiamplitude-driven annular transducer array consisting of a few elements. *Jpn. J. Appl. Phys.* **38**, 3080 (1999).

32. Fjield, T., Fan, X. & Hynynen, K. A parametric study of the concentric-ring transducer design for mri guided ultrasound surgery. *J. Acoust. Soc. Am.* **100**, 1220 (1996).

33. Chillara, V. K., Pantea, C. & Sinha, D. N. Low-frequency ultrasonic Bessel-like collimated beam generation from radial modes of piezoelectric transducers. *Applied Physics Letters* **110**, 064101 (2017).

34. Burckhardt, C., Hoffmann, H. & Grandchamp, P.-A. Ultrasound axicon: A device for focusing over a large depth. *The Journal of the Acoustical Society of America* **54**, 1628–1630 (1973).

35. Foster, F., Patterson, M., Arditi, M. & Hunt, J. The conical scanner: a two transducer ultrasound scatter imaging technique. *Ultrasonic imaging* **3**, 62–82 (1981).

36. McLeod, J. H. The axicon: A new type of optical element. *J. Opt. Soc. Am.* **44**, 592 (1954).

37. Arlt, J. & Dholakia, K. Generation of high-order Bessel beams by use of an axicon. *Optics Communications* **177**, 297–301 (2000).

38. Golub, I. Fresnel axicon. *Optics letters* **31**, 1890–1892 (2006).

39. Lirette, R. & Mobley, J. Broadband wave packet dynamics of minimally diffractive ultrasonic fields from axicon and stepped fraxicon lenses. *The Journal of the Acoustical Society of America* **146**, 103–108 (2019).

40. Jiménez, N. *et al.* Acoustic Bessel-like beam formation by an axisymmetric grating. *Europhys. Lett.* **106**, 24005 (2014).

41. Xu, Z., Xu, W., Qian, M., Cheng, Q. & Liu, X. A flat acoustic lens to generate a Bessel-like beam. *Ultrasonics* **80**, 66–71 (2017).

42. Li, Y., Liang, B., Gu, Z.-m., Zou, X.-y. & Cheng, J.-c. Reflected wavefront manipulation based on ultrathin planar acoustic metasurfaces. *Scientific Reports* **3**, 2546 (2013).

43. Nye, J. & Berry, M. Dislocations in wave trains. *Proc. R. Soc. London, Ser. A* **336**, 165–190 (1974).

44. Jiménez, N. *et al.* Formation of high-order acoustic Bessel beams by spiral diffraction gratings. *Physical Review E* **94**, 053004 (2016).

45. Wang, T. *et al.* Particle manipulation with acoustic vortex beam induced by a brass plate with spiral shape structure. *Applied Physics Letters* **109**, 123506 (2016).

46. Jia, Y.-R., Wei, Q., Wu, D.-J., Xu, Z. & Liu, X.-J. Generation of fractional acoustic vortex with a discrete archimedean spiral structure plate. *Applied Physics Letters* **112**, 173501 (2018).

47. Jiménez, N., Romero-García, V., García-Raffi, L. M., Camarena, F. & Staliunas, K. Sharp acoustic vortex focusing by fresnel-spiral zone plates. *Applied Physics Letters* **112**, 204101 (2018).

48. Baudoin, M. *et al.* Folding a focalized acoustical vortex on a flat holographic transducer: miniaturized selective acoustical tweezers. *Science advances* **5**, eaav1967 (2019).

49. Muelas-Hurtado, R. D., Ealo, J. L., Pazos-Ospina, J. F. & Volke-Sepúlveda, K. Acoustic analysis of a broadband spiral source for the simultaneous generation of multiple Bessel vortices in air. *The Journal of the Acoustical Society of America* **144**, 3252–3261 (2018).

50. Muelas-Hurtado, R. D., Ealo, J. L., Pazos-Ospina, J. F. & Volke-Sepúlveda, K. Generation of multiple vortex beam by means of active diffraction gratings. *Applied Physics Letters* **112**, 084101 (2018).

51. Wunenburger, R., Lozano, J. I. V. & Brasselet, E. Acoustic orbital angular momentum transfer to matter by chiral scattering. *New Journal of Physics* **17**, 103022 (2015).

52. Terzi, M., Tsysar, S., Yuldashev, P., Karzova, M. & Sapozhnikov, O. Generation of a vortex ultrasonic beam with a phase plate with an angular dependence of the thickness. *Moscow University Physics Bulletin* **72**, 61–67 (2017).

53. Hefner, B. T. & Marston, P. L. An acoustical helicoidal wave transducer with applications for the alignment of ultrasonic and underwater systems. *Jour. Acous. Soc. Am.* **106**, 3313–3316 (1999).

54. Ealo, J. L., Prieto, J. C. & Seco, F. Airborne ultrasonic vortex generation using flexible ferroelectrets. *IEEE transactions on ultrasonics, ferroelectrics, and frequency control* **58**, 1651–1657 (2011).

55. Naify, C. J. *et al.* Generation of topologically diverse acoustic vortex beams using a compact metamaterial aperture. *Applied Physics Letters* **108**, 223503 (2016).

56. Ye, L. *et al.* Making sound vortices by metasurfaces. *AIP Advances* **6**, 085007 (2016).

57. Jiang, X., Li, Y., Liang, B., Cheng, J.-c. & Zhang, L. Convert acoustic resonances to orbital angular momentum. *Physical review letters* **117**, 034301 (2016).

58. Esfahlani, H., Lissek, H. & Mosig, J. R. Generation of acoustic helical wavefronts using metasurfaces. *Physical Review B* **95**, 024312 (2017).

59. Jiménez-Gambín, S., Jiménez, N., Benlloch, J. M. & Camarena, F. Holograms to focus arbitrary ultrasonic fields through the skull. *Physical Review Applied* **12**, 014016 (2019).

60. Maimbourg, G., Houdouin, A., Deffieux, T., Tanter, M. & Aubry, J.-F. 3d-printed adaptive acoustic lens as a disruptive technology for transcranial ultrasound therapy using single-element transducers. *Physics in Medicine & Biology* **63**, 025026 (2018).

61. Ferri, M. *et al.* On the evaluation of the suitability of the materials used to 3d print holographic acoustic lenses to correct transcranial focused ultrasound aberrations. *Polymers* **11**, 1521 (2019).

62. Melde, K., Mark, A. G., Qiu, T. & Fischer, P. Holograms for acoustics. *Nature* **537**, 518 (2016).

63. Brown, M. D., Cox, B. T. & Treeby, B. E. Design of multi-frequency acoustic kinoforms. *Applied Physics Letters* **111**, 244101 (2017).

64. Brown, M., Nikitichev, D., Treeby, B. & Cox, B. Generating arbitrary ultrasound fields with tailored optoacoustic surface profiles. *Applied Physics Letters* **110**, 094102 (2017).

65. Zhu, Y. *et al.* Fine manipulation of sound via lossy metamaterials with independent and arbitrary reflection amplitude and phase. *Nature communications* **9**, 1632 (2018).

66. Brown, M. D. Phase and amplitude modulation with acoustic holograms. *Applied Physics Letters* **115**, 053701 (2019).

67. Jiménez, N., Romero-García, V., Pagneux, V. & Groby, J.-P. Quasiperfect absorption by subwavelength acoustic panels in transmission using accumulation of resonances due to slow sound. *Physical Review B* **95**, 014205 (2017).

68. Tsang, P. W. M. & Poon, T.-C. Novel method for converting digital fresnel hologram to phase-only hologram based on bidirectional error diffusion. *Optics Express* **21**, 23680–23686 (2013).

69. Soret, J. Ueber die durch kreisgitter erzeugten diffractionsphänomene. *Annalen der Physik* **232**, 99–113 (1875).

70. Turunen, J., Vasara, A. & Friberg, A. T. Holographic generation of diffraction-free beams. *Applied Optics* **27**, 3959–3962 (1988).

71. Vasara, A., Turunen, J. & Friberg, A. T. Realization of general nondiffracting beams with computer-generated holograms. *JOSA A* **6**, 1748–1754 (1989).

72. Cunningham, K. B. & Hamilton, M. F. Bessel beams of finite amplitude in absorbing fluids. *J. Acous. Soc. Am.* **108**, 519 (2000).

73. Ding, D. & y. Lu, J. Higher-order harmonics of limited diffraction Bessel beams. *J. Acous. Soc. Am.* **107**, 1212 (2000).

74. Thomas, J.-L. & Marchiano, R. Pseudo angular momentum and topological charge conservation for nonlinear acoustical vortices. *Phys. Rev. Lett.* **91**, 244302 (2003).

75. Volke-Sepúlveda, K., Santillán, A. O. & Boullosa, R. R. Transfer of angular momentum to matter from acoustical vortices in free space. *Phys. Rev. Lett.* **100**, 024302 (2008).

76. Skeldon, K., Wilson, C., Edgar, M. & Padgett, M. An acoustic spanner and its associated rotational Doppler shift. *New J. Phys.* **10**, 013018 (2008).

77. Wu, J. Acoustical tweezers. *J. Acoust. Soc. Am.* **89**, 2140–2143 (1991).

78. Zhang, L. & Marston, P. L. Angular momentum flux of nonparaxial acoustic vortex beams and torques on axisymmetric objects. *Physical Review E* **84**, 065601 (2011).

79. Yoon, C., Kang, B. J., Lee, C., Kim, H. H. & Shung, K. K. Multi-particle trapping and manipulation by a high-frequency array transducer. *Appl. Phys. Lett.* **105**, 214103 (2014).

80. Marzo, A. *et al.* Holographic acoustic elements for manipulation of levitated objects. *Nat. Commun.* **6** (2015).

81. Blackstock, D. T. *Fundamentals of physical acoustics* (John Wiley & Sons, 2000).

82. Treeby, B. E. & Cox, B. Modeling power law absorption and dispersion for acoustic propagation using the fractional laplacian. *The Journal of the Acoustical Society of America* **127**, 2741–2748 (2010).

83. Treeby, B. E., Jaros, J., Rendell, A. P. & Cox, B. Modeling nonlinear ultrasound propagation in heterogeneous media with power law absorption using a k-space pseudospectral method. *The Journal of the Acoustical Society of America* **131**, 4324–4336 (2012).

84. Jiménez, N. *et al.* Time-domain simulation of ultrasound propagation in a tissue-like medium based on the resolution of the nonlinear acoustic constitutive relations. *Acta Acustica united with Acustica* **102**, 876–892 (2016).

Acknowledgements

This work was supported by the Spanish Ministry of Economy and Innovation (MINECO) through Project TEC2016-80976-R. NJ and SJ acknowledge financial support from Generalitat Valenciana through grants APOSTD/2017/042, ACIF/2017/045 and GV/2018/11. FC acknowledges financial support from Agència Valenciana de la Innovació through grant INNCON00/18/9 and European Regional Development Fund (IDIFEDER/2018/022).

Author contributions statement

NJ and FC conceived the idea and conducted the theoretical modelling; SJG, NJ, and FC performed the numerical simulations and experiments; and NJ, SJG, JMB, FC wrote the manuscript. All authors reviewed the manuscript.

Additional information

Competing interests The authors declare no competing interests.
